# Lessons from the Field Beyond the Numbers: Narratives of Professionals on Women who Experienced Severe Maternal Morbidity

**DOI:** 10.1055/s-0039-1688833

**Published:** 2019-06

**Authors:** Carina Fernanda Robles Angelini, Rodolfo de Carvalho Pacagnella, Carla Silveira, Carla Betina Andreucci, Elton Carlos Ferreira, Juliana Pereira Santos, Dulce Maria Toledo Zanardi, Mary Angela Parpinelli, Maria Laura Costa, José Guilherme Cecatti

**Affiliations:** 1 Department of Gynecology and Obstetrics, Faculdade de Medicina, Universidade Estadual de Campinas, Campinas, SP, Brazil; 2 Department of Medicine, Faculdade de Medicina, Universidade Federal de São Carlos, São Carlos, SP, Brazil

**Keywords:** personal narratives, childbirth, health personnel, mothers, postpartum period, narrativas pessoais, nascimento, profissionais de saúde, período pós-parto

## Abstract

**Objective** Several factors might affect the health and the quality of life of women who had a severe maternal morbidity (SMM) or a maternal near-miss (MNM) episode. The objective of the present study was to explore the perspectives of the professionals on the repercussions of SMM or of MNM after interviewing women who survived such episodes.

**Method** Selected cases that captured the attention of professionals were reported. The professionals built individually 10 narratives, which were analyzed with the technique of content analysis.

**Results** According to the perspectives of the professionals, women surviving a severe maternal condition and their families experienced clinical and psychosocial consequences. Some cases portrayed the intense psychological distress in mourning for the loss of the fetus or of their reproductive capacity and changes in family dynamics generating emotional overload, depression, and gender violence.

**Conclusion** The analysis of narratives may offer an idea on the complexity of the perception of care by professionals and on the need for an interdisciplinary follow-up of women surviving an SMM or an MNM episode.

## Abstract (pt)

**Resumo:**

**Objetivo** Diversos fatores podem afetar a saúde e a qualidade de vida das mulheres que tiveram um episódio de morbidade materna grave (MMG) ou near-miss materno (NMM). O objetivo do presente estudo foi explorar as perspectivas dos profissionais sobre as repercussões da MMG ou do NMM após terem entrevistados mulheres que sobreviveram a um desses episódios.

**Métodos** Casos selecionados que chamaram a atenção dos profissionais foram relatados. Estes profissionais construíram individualmente 10 narrativas, que foram analisadas com a técnica de análise de conteúdo.

**Resultados** Segundo as perspectivas dos profissionais, as mulheres que sobreviveram a uma condição materna grave e suas famílias vivenciaram consequências clínicas e psicológicas. Alguns casos relataram um intenso estresse psicológico no luto pela perda do feto ou de sua capacidade reprodutiva e de mudanças da dinâmica familiar, gerando sobrecarga emocional, depressão e violência de gênero.

**Conclusão** A análise das narrativas pode oferecer uma ideia sobre a complexidade da percepção do cuidado de profissionais e sobre a necessidade de um seguimento interdisciplinar das mulheres sobreviventes de um episódio de MMG ou de NMM.

## Introduction

Recent data indicate a considerable reduction of maternal mortality in Brazil. This decrease is partly due to the qualification of obstetric care offered to the health of the women and of the emergency facilities.^1^ However, it is also necessary to seek beyond surviving cases. This is a challenging task, because little is already known about the long-term repercussions of maternal complications on the mental health and on the quality of life of these postpartum women.^2^ ^3^ In addition, information on long-term repercussions of severe maternal morbidity (SMM) has not yet been fully explored. Available data are scarce; however, they are necessary to improve health care and the prevention of damage to surviving women.^3^ ^4^ ^5^ ^6^ Lack of knowledge might hinder the desirable convergence between the reduction in maternal deaths and the decrease in severe complications of pregnancy.^7^

Recently, studies have been carried out to understand and assess risk factors and potential strategies for the prevention of maternal near-miss.^8^ The combination of severe life-threatening complications might trigger intense physical and psychological distress and might culminate in post-traumatic stress disorder (PTSD), not limited to the postpartum period, among other problems.^7^ ^9^ ^10^ ^11^

The assessment of pregnancy repercussions usually does not extend beyond the postpartum visit at 6 weeks after delivery. Nevertheless, women suffering from severe maternal morbidity might continue to have long-term negative consequences of this episode.^4^ Survivors of obstetric complications are more physically and socially vulnerable. They are also more prone to develop postpartum mental issues, such as depression and anxiety.^12^ Fear of death, loss of hope, concerns about possible upcoming surgical procedures, memory lapses, mourning for the loss of the baby or of the reproductive capacity because of hysterectomy, feelings of loss of female identity, among others, have already been recognized.^7^ ^13^

These results underline the need to make efforts in both directions: first, to reduce maternal losses; and second, to provide care beyond the postpartum period, considering that ∼ 9.5 million women suffer from complications during pregnancy, childbirth, or the postpartum period annually and survive.^14^

Testimonies by female survivors are currently used to understand and identify management problems and other determining factors of the health-disease process in women.^4^ ^7^ However, beyond listening to the women, it is also fundamental to understand the difficulties of care from the perspective of the professionals. Therefore, the purpose of the current study was to understand the perceptions of the health care professionals of these consequences and of the health care conditions offered to postpartum women who have suffered an SMM or a maternal near-miss (MNM) episode.

## Methods

This is a descriptive qualitative study emerging from the narratives of health professionals involved in a retrospective cohort study aimed to understand the long-term repercussions of SMM and MNM on the various domains of the lives of the women, in a multidimensional way.^15^ The researchers (obstetricians, pediatricians, nurses, and psychologists) wrote the narratives during interviews with women who have survived an SMM. In the current analysis, we have selected some stories that drew special attention of professionals at the time the interviews were performed.

The women were contacted by telephone and invited to participate in the study. The perception of health status, reproductive history, quality of life, PTSD, sexual dysfunction, multiple disabilities and functioning, as well as growth and development conditions of the child were evaluated using specific tools for each assessment.^2^ ^15^

During the training for the interviews, the health professionals were instructed to describe the most interesting cases that called their attention, with a written report that should be a narrative from their own perspective. The instructions on how each narrative should be written were not predefined. The main orientation for the interviewers was for them to "listen beyond the questionnaires" and to register all that affected them while listening to the women during the interviews.

The narratives were different in form as they were the result of a spontaneous record of what had affected each professional. We have decided to analyze the narratives considering that they are important tools for health professionals to express their perception of their daily work, including emotional issues. Thus, they transcribed cases and life histories that most captured their attention. The reports gave important information from the perspective of each professional involved in the data collection, similarly to the process of describing and reporting narratives in their clinical practice.

The process of analysis was based on the Bardin content analysis technique,^16^ which offers the possibility of a qualitative or quantitative exploration of messages and information on various documents and texts. It helps to reinterpret messages and to achieve comprehension of their meanings at a level that goes beyond the common reading. Therefore, in the present study, we have adopted the qualitative content analysis technique. All of the narratives were analyzed as they composed a diversity of clinical conditions and represented different forms of understanding the topic by different professionals. Each narrative was defined as a unit of analysis that would be submitted to interpretation, because it contained information with a complete significance in itself.

For the analysis, we used the following steps: presentation and reading of the reports for the group of researchers; coding records as narratives 1 to 10; several careful readings of all of the narratives to identify underlying themes; and finally, the thematic analysis of each narrative. Categorization was performed by similarity and analogy, and it was not defined a priori, but emerged from the reading and the proximity of the records.^16^ Finally, we allocated the complete description of each record produced by the health professionals to defined categories. The categories were built during the analysis, not to generalize the results nor to test hypotheses, but in order to understand perceptions of the professionals on the health status and on the challenges of the women for the management of these conditions. The categories were titled arbitrarily, but the literature on maternal morbidity was pivotal.

The research proposal generating these records was approved by the Institutional Review Board (letter 233/2009). After information on the study proposal, all of the participants signed a written consent form. Their names were kept confidential.

## Results and Discussions

A total of 10 narratives was selected for analysis. They illustrate the perception of health professionals on the clinical, emotional, and psychosocial repercussions of SMM and MNM on the lives of the women. It was assumed that the narratives of health professionals could bring singularities to each one, and that they would produce metahistories of disorders, thus promoting a new understanding. The professionals needed to understand the significance of the disorder from the perspective of the woman to compose their narratives based on the symptoms of the patient.^17^ Qualitative analysis did not permit generalization of the results, but it allowed the exploration and the understanding of certain specific situations. Indeed, there was no intention of generalizing the results.^18^

Based on the definition of the 10 units of analysis, after a thorough reading of the material, 4 categories emerged:

**1- The need of longitudinal care beyond the postpartum period:**

**Narrative 5**

*MMCR, age 42, was very anxious when we met. I remember that she was worried about the type of questions I would ask, because she could not remember things that had happened. [...] She underwent a surgery because she had an ectopic pregnancy and one of her tubes had ruptured. She had her first pregnancy at 24 years old that resulted in a spontaneous abortion. She did not know about the pregnancy and only remembered waking up at the intensive care unit (ICU). The doctor told her that the baby was dead but she knew nothing about it. [...] After 3 years, she got pregnant. She cried a lot when she told her story. A year later, bleeding began and she underwent a new surgery for the removal of her uterus, which was full of fibroids.*

**Narrative 7**

*ECA, age 40, member of an evangelical church. She has only one baby but had four pregnancies. In her 1^st^ pregnancy, she had an abortion at 36 years old. In the 2^nd^ pregnancy, she had a baby who is 3 years old today. The 3^rd^ pregnancy had no fetus [...] I explained that, eventually, women might have a fertilized egg connected to the uterine walls but no development of an embryo. I told her that she and her husband were not guilty. The 4^th^ pregnancy was a spontaneous abortion. During the interview, she cried a lot [...] I offered her psychological assistance, which she accepted, although she had never thought about it. Finally, I gave her the support to go on.*

Ectopic pregnancy is a severe obstetric condition, and the leading cause of maternal deaths in the 1^st^ trimester of pregnancy; it might be diagnosed early and managed conservatively,^19^ and might also result in infertility.^20^ The text showed the emotional impact experienced by the woman. In a similar way, narrative 7 produced the clinical history of a woman who had repeated abortions, followed by a successful term pregnancy. From a medical and reproductive point of view, abortion is considered a clinically common event. It might be the reason why the event is not highly valued by both health care providers and by society.^21^

Testimonies on late consequences of ectopic pregnancy and abortion were shown, including emotional aspects of the woman, and difficulty in understanding what happened to her body and health. Persistent doubts about possible causes of abortions, and the challenge in explaining to women what actually happened, might generate equivocal beliefs in women, such as fantasies of guilty. As a result, the woman might become emotionally vulnerable to stress,^21^ to sadness, and to feelings of helplessness.^22^

The emotional impact generated by perinatal death might cause a state of deep mourning, affecting the women for a long period. This could generate marital conflicts, persistent depression, and social isolation. In contrast, in narrative 1, shown below, the woman had received longitudinal care where she was admitted during her 1^st^ pregnancy. The hypertensive condition seemed to be a protective factor for her in terms of continued medical care.

The health professional described complications during the pregnancy, resulting in fetal death. Her needs were met in a timely way, since longitudinal care was offered. Health care was not provided only in an acute emergency event. The situation was perceived as a “relief” by the health professional describing the narrative, because a residual morbid condition allowed the woman to receive better medical care.

***Narrative 1***

*A 38-year-old woman, obese, with chronic hypertension. She had her 1^st^ delivery at the same institution 2 years before, when she developed a superimposed preeclampsia and fetal growth restriction, with admission at 26 weeks of gestation for fetal monitoring. An intrauterine fetal death occurred at 28 weeks. She underwent labor induction and had a vaginal delivery. After ∼ 6 months postpartum, she had an open myomectomy for removal of a large uterine fibroid. Today, she is still under monitoring in the institution to control blood pressure and is preparing herself for a new pregnancy. She arrived smiling, confident, and humorous at the outpatient clinic. She was well-dressed and very peaceful about a new pregnancy. During the visit, she did not show any trace of anxiety or fear that a new SMM could occur during a future pregnancy. She barely spoke about the loss of her 1^st^ child, a boy. There was sadness in her prepared speech and attitude, but she clearly preferred to maintain a positive outlook. A partner with a stable financial condition supported her. She made a few questions and walked away looking very satisfied with the appointment, despite the hardships she went through.*

There is evidence that medical and psychological care for a woman suffering from early reproductive loss might have a significant effect on her experience and physical/emotional recovery.^21^ Furthermore, professional health care extended to the family nucleus is an important resource to reduce the period of mourning and its negative consequences.^23^ However, health professionals do not always acknowledge this, once early reproductive loss is interpreted as a common event and the preservation of the life of the woman might be felt as the end of the action.^24^

Health professionals find it difficult to approach emotionally painful situations such as the loss of a baby.^25^ Adequate management with friendly sensitive treatment influences how the woman experiences this event.^10^ ^26^ Therefore, preparing and providing health professionals with tools for dealing with their own emotions is indicated as a necessary resource for the quality of care.^24^ Furthermore, the approach to female mental health should not be limited to specialized facilities or to professionals such as psychiatrists or psychologists, because they are not always available.

**2- The impact of severe maternal morbidity and maternal near-miss on the mental health of women and management difficulties in a woman health care facility:**

**Narrative 6**

*A 30-year-old woman, who first became pregnant during adolescence and had an elective cesarean section. After a few years, she got married to another partner, and got spontaneously pregnant with twins. This pregnancy evolved with a twin-to-twin transfusion syndrome (TTTS) in the 5^th^ month of pregnancy and intrauterine loss of both children. Birth was induced. She stated that she became very sad and tearful, requiring treatment with psychotropic drugs and help from a mental health professional. After 2 years, she presented with a new spontaneous pregnancy, when she was enrolled in the study. It was another twin pregnancy, also with progression to severe TTTS between the 4^th^ and 5^th^ month. She was hospitalized during several days for respiratory failure because of polyhydramnios of the hydropic fetus, which was repeatedly drained. She claimed that cesarean section was indicated at 26 weeks of gestation and that there was little hope for survival of the newborns. The babies were born, admitted to the neonatal intensive care unit (NICU), where they spent several months. During the admission to the NICU, she became clinically depressed. Now, it has been 2 years since this pregnancy. Even though both children survived without complications, the patient refers mood swings, tearfulness, insomnia, panic attacks, and barely leaves the house with fear of the world around her. She refers a worse quality of life since the last pregnancy. She almost has no sex and experiences a troublesome marital relationship.*

***Narrative 9***

*H., 36 years old, full higher education, circus acrobat, married. She underwent a cesarean section in her 1^st^ pregnancy 3 years earlier because of “lack of dilation”. The patient reported that she was ready for vaginal delivery but “was in labor for 15 days and did not dilate”. Her prenatal care was in a private health facility and one of the physicians was her sister “who did everything possible for her to deliver vaginally”. In her 2^nd^ pregnancy, she started bleeding in the 5^th^ month and was referred to the institution for follow-up. At that moment, she was using supplemental health insurance for prenatal care. She was then diagnosed with total central placenta previa and placental percretism. She also developed severe preeclampsia, requiring the use of magnesium sulfate and prolonged hospitalization. She underwent cesarean section at 37 weeks of gestation, with uterine artery embolization and subsequent postpartum hysterectomy. She was admitted to the ICU and received transfusion therapy. It has been 8 months since childbirth, and she is still breastfeeding. She cried a lot during the visit. She feels mutilated for not having a uterus and not being able to deliver anymore. She almost never has sex. She refers that the abdominal scar is horrible and disfigures her body. She was unable to lose the 10 kg that she gained during pregnancy, so she feels diminished concerning physical aesthetics, an area of great value to her. She is unable to practice all the activities of her presentation and her gym clothes no longer fit her. At the same time, she refused to undergo psychological follow-up. She considered that she has no right to be sad, because she is alive, has healthy children and a loving husband.*

***Narrative 3***

*AS, 38 years old, in her 2nd pregnancy, presented with bleeding in the 1^st^ and 2nd trimesters. She was diagnosed with total placenta previa and placenta percreta. She was referred to the institution, where she underwent an elective cesarean section at 32 weeks of gestation, uterine artery embolization, and subtotal hysterectomy. During the late postoperative period, she developed a vesicovaginal fistula, which required medical and surgical treatment. The urological follow-up lasted for several months, which compromised her quality of life, taking care of her premature boy, and breastfeeding.*

In the narratives above, the professionals described potentially fatal obstetric and clinical complications, in which the women survived. The opportunity to know histories based on narratives of women provides health professionals with the chance to understand the facts biased toward success. These women were alive, in spite of everything.^27^ In contrast, studies have shown that even after hospital discharge, survivors of an MNM event were more vulnerable to physical, psychological, and social debilitating consequences up to 1 year after childbirth.^5^ ^27^ ^28^ ^29^ They might even have the potential risk of developing PTSD,^9^ and postpartum depression.^28^

In the three narratives above, opportune obstetric interventions were noted, as well as the action responsible for the survival of these women. Nevertheless, medical care provided during hospitalization appeared as insufficient to avoid the emergence or maintenance of suffering and mental illness in these women after discharge. It is known that mental health of a woman might be affected not only by a particular event, but also by the cumulative effect of various circumstances in life.^5^

In narrative 6, the neonatal outcome was positive, if compared with the previous pregnancy, in which intrauterine fetal death occurred. In narrative 9, despite the complications, the baby was also born alive. The literature indicates that the successful experience of giving birth to a live baby after a near-miss episode might be a protective factor for the mental health of a woman.^5^ Nevertheless, each woman attributes singular meaning to the lost pregnancy. Difficulties generated by family or marital tensions, devaluation of body image, sexual, economic, and social difficulties, among others, might compromise the recovery of health conditions desired by women.

The psychiatric impact of SMM on the lives of women is uncertain, because of multiple factors that might be associated with mental disorders. However, recent studies have suggested that there might be a potential relationship between SMM and PTSD symptoms.^9^ These symptoms might impair the quality of affective and social relationships,^11^ and have negative repercussions on the well-being of a woman.^28^

In a recent review of qualitative studies on the perception of patients of SMM,^10^ it was concluded that women frequently affected by comorbid conditions required health care after discharge. The negative impact of SMM on mental health might be expressed a long time after the postpartum period. However, the quantitative results of the current study, although showing negative impacts on maternal functioning, sexual health and quality of life,^29^ ^30^ ^31^ were not able to identify an important impact on the occurrence of PTSD among women experiencing SMM at least up to 5 years after the event.^32^

**3- The woman in a vulnerable state and the challenges of daily practice in health care facilities:**

**Narrative 10**

*It was a case of a diabetic mother, with complications because of severe preeclampsia in her unplanned 1^st^ pregnancy. It seems that she was adequately referred to the University hospital. In addition to ICU admission and all clinical complications, there were no major consequences. She wanted her next pregnancy, was not afraid and had no further complications. Her 2nd prenatal care was not even classified as high-risk and received no referral. Thank God she had no complications on her 2^nd^ pregnancy (I cannot explain how!). But now she is completely involved in taking care of her kids with no time to control her diabetes or to attend routine medical appointments. She is not feeling well. Her glucose levels are very high and she has symptoms of neuromyopathy. She uses no contraceptive method. While we were filling all the answers to the research questionnaires, she burst into tears… she felt she had the right. At that moment, she realized all the things she had been through and all that was missing in her life. She was happy for the boys, but also worried, maybe for the first time. She promised to try hard to control her diet and medication. She promised to schedule an appointment with her endocrinologist and another with her gynecologist. Promises… However, this was ONE appointment… I might not ever know what happens to this nice woman… unless I see her again during her next pregnancy… I hope not as a new near-miss event.*

**Narrative 8**

*EFC, 23 years old, epileptic. She could not enlist the help of her partner – neither financial nor emotional support (they never lived together). In late pregnancy, she was diagnosed with depression, persisting until the present date. Last year, she had to leave her sister, who was going through serious financial difficulties. By that time, she brought her mother and son with her. She attempted suicide in July 2011 after quarrelling with her sister. She was hospitalized for 30 days in a psychiatric clinic. Now she reports partial improvement of depression, especially after leaving home. She did psychiatric and neurological monitoring and described important improvement in her medical status. During an acute phase of depression, she also stated that her mother was responsible for taking care of her son. The woman said that her ex-husband frequently bothers her, threatening to demand custody of their child.*

**Narrative 4**

*CLT is 35 years old, currently living in her niece's house. Her partner lives elsewhere. She is jobless and still using drugs. Talking to her niece on the first contact, the interviewer found out that CLT is a homeless drug user and a wanderer. CLT told me that she is currently smoking and using crack but had also used "oxy". I asked her what that drug was made of. She supposed that it is a mixture of crack and burnt car oil because the small rocks are black. She noticed that her street friends looked like "animals" after using that substance. She confessed that she and her partner could live without using drugs for up to 2 weeks. However, they had frequent relapses and did use drugs again. Her partner was in ill health. He had tuberculosis and was rather weak. I suspected that he was HIV positive. CLT is quite lucid, finished high school and had an appointment with the general practitioner, plus blood tests at the health unit near her house. At present, she is followed in an outpatient clinic for mental health because she is depressed. She said that, in recent years, she had been living on the streets, where she became a prostitute. She was repeatedly a victim of sexual violence, even attacked by military police. She had six pregnancies, one normal delivery, two cesareans, and three abortions. Interestingly, she wanted four children in her life and one more after her last son. In her 1^st^ pregnancy, fetal death occurred. In her last pregnancy, she used crack all the time. She has three living children, but does not have custody of any child. She demonstrated deep love for her youngest child and said that she intends to get better in the future to have custody of the children. Two sons are from the current partner. I offered psychiatric outpatient care at the institution for patients addicted to chemical substances, but at the time she had no interest in treatment. I asked if we could help her in any way and she answered no. I finished the interview by making myself available, in case she needed any more help in the future. Participants usually receive some money for covering expenses. However, before the interview, the caregiver asked me not to tell her about the money, because she would use it to buy drugs. As CLT was leaving the room, she asked about her child's growth, if it was normal according to the pediatrician. Then she went to meet her son and her niece, who had been waiting at the entrance of the building.*

In these narratives, health professionals not only informed about the fragile health conditions of the women, but also how vulnerability in health emerges and/or can be aggravated over time. Women in vulnerable conditions are more exposed to illnesses and have a higher risk of dying. In addition, resources must be taken into account, such as the basic conditions prior to the disease; the social, economic, and psychological aspects, as well as any situation that exposes or protects a person from morbid situations.

From this perspective, in the three narratives presented, it was possible to identify different conditions of vulnerability in these women. In narrative 10, the clinical history was not biased toward successful health interventions. According to the professional, the fact that the woman was still alive was attributed to luck and to a supernatural force. In the three narratives presented, professionals evidenced emotional and social vulnerabilities in the women who were exposed. The narratives indicated that health interventions for these women needed to be extended beyond the time for disease control to minimize the vulnerable condition of the patient. The reason is that social issues of gender, cognition, and emotional aspects may keep these patients vulnerable and at a higher risk of death.

In narrative 10, the patient was expected to follow the instructions and seek health facilities, because she is at risk of a new pregnancy and her condition demands care. Similarly, in narrative 4, the health professional used interventions to understand the health conditions of the woman. However, these interventions were not very effective when he tried to include the patient in a facility considered most appropriate for her at the time. Despite a history of six pregnancies and three abortions, the practice of prostitution and the exposure to contamination by infectious-contagious diseases and violence, referral to a family planning program was not performed. The patient was referred to a specialized mental health facility.

Furthermore, the search for and compliance with treatments are not simple processes and involve the capacity to understand the problem, to incorporate knowledge, and to transform behavior based on the rapport established between health professionals and patients. Although morbidity may result from biological processes, another factor that makes management of these narratives more difficult is that women respond to these events in a manner that is not unique or biologically determined.^17^

Even if health facilities are available, health care models focused on the management of acute conditions and the lack of longitudinal care may contribute to increased maternal morbidity and mortality.^27^ Furthermore, the medical diagnosis of obstetric complications and the manner in which a woman understands and recognizes this diagnosis do not always agree. The medical point of view is not always the same as that of a woman. This can determine seeking behavior and compliance with treatment.^28^

Despite new guidelines and international recommendations, mental health care actions have still not been incorporated in the routine practice of specialized health service for women in Brazil. When pregnant women or those in the postpartum period become mentally ill, they are referred to a specialized mental health facility, which does not offer conditions to care for specific female issues.

**4- Violence against women, its impact on health, and complexity of management in health services:**

**Narrative 2**

*A 34 year-old-woman. Her 1^st^ pregnancy was diagnosed with total central placenta previa, which was treated with an elective cesarean section. At the immediate postoperative period, the patient developed postpartum hemorrhage and underwent a new laparotomy with subtotal hysterectomy, need for massive blood transfusion, and admission to the ICU. She had a larger family expectation. The marital relationship was bad, the couple was about to split when she got pregnant and decided to maintain the marriage for the benefit of the child to be born. Before the pregnancy, she was also a victim of aggression by the partner, besides having suffered repeated betrayals. After the hysterectomy, she felt less feminine and regretted the impossibility of further pregnancies. The marital relationship remains poor, although the attacks today are psychological and not physical. The verbal abuse to which her partner submits her includes references to a foul vaginal odor, not recognized either by the woman herself or by any of the gynecologists that had examined her after childbirth. They do not have intercourse often, and her husband frequently repeats that she is no longer attractive to him. Eventually, when they consummate their relation, he complains about “her vaginal odor”, using degrading terms and pimps. She wept during the query, desolated by what occurred during the birth of her daughter, and by being tied in a conjugal relationship of aggression and with no prospects. A mental health professional has followed her, but she had little improvement of the clinical depression. She does not take any medication. She refers that she often cries, but she appears well cared for and well nourished.*

In narrative 2, the history of violence suffered in the domestic setting with acts of aggression displayed by the intimate partner and the obstetric outcome culminating in hysterectomy are highlighted. Both put the woman in a fragile position. Female infertility may generate negative social consequences, especially in social settings in which gender identities and values are defined by the fertility capacity of a woman.^5^ ^33^ In this narrative, the woman appears as someone who feels less feminine with the impossibility of new pregnancies, feeling incapable of satisfying her partner sexually. Nevertheless, she remains connected to him and suffers violence. Psychological and sexual violence associated with deficiencies in women resulting from obstetric complications may increase their vulnerability.^34^

Dealing with the topic of violence is difficult, owing to its complexity and to the understanding that it is an intimate subject. It demands the ability of health professionals who are not always skilled. They often have to deal with what is not said and what one is afraid to say because it may be considered rejected, forbidden or embarrassing.^35^ Therefore, they must approach these women and establish a bond, turning something nonexistent (and unmanageable) into something real.

The approach to violence against women in health care settings is challenging. In addition, there is a demand for long-term intersectoral actions that are difficult to put into practice when health care models prioritize the approach to disease symptoms and do not have resources to address the problem.^36^

For this purpose, the organization of interdisciplinary health actions in follow-up models should not be directed only at investigating and managing diseases, but must also be concerned with the approach to situations that increase the risk and vulnerability of these women and with the impact on the loss of their quality of life. These are the main reasons why, recently, the World Health Organization (WHO) is recommending a more integrated approach for dealing with pregnant or postpartum women experiencing not only maternal morbidities but also mental or social impairments.^37^

## Conclusion

Narratives provided information about long-term repercussions of SMM and/or MNM, according to the perception of health professionals. They presented the singularities of each case. In addition, they showed how long-term maternal complications extended beyond the postpartum period, affected the health and lives of women, along with other aspects. Complex life histories emerged, narrated beyond the clinical symptoms that are usually investigated in the clinical practice. Active listening and biomedical aspects in the clinical practice provided health professionals with life histories and life narratives. These professionals met another dimension in the health-disease process. Thus, from their perspective, these cases showed that the repercussions of SMM are not restricted only to the pregnancy and postpartum period, and that they demand longitudinal and interdisciplinary care beyond the full postpartum period. These cases indicated the importance of careful listening.
